# The association of *MPO* gene promoter polymorphisms with Alzheimer’s disease risk in Chinese Han population

**DOI:** 10.18632/oncotarget.22330

**Published:** 2017-11-06

**Authors:** Wenzhen Ji, Yu Zhang

**Affiliations:** ^1^ Department of Neurology, Tianjin Huanhu Hospital, Tianjin Key Laboratory of Cerebrovascular and Neurodegenerative Diseases, Tianjin 300000, China; ^2^ Division of Medical Affairs, Tianjin Huanhu Hospital, Tianjin Key Laboratory of Cerebrovascular and Neurodegenerative Diseases, Tianjin 300000, China

**Keywords:** Alzheimer's disease, *MPO*, polymorphisms, plasma concentration

## Abstract

**Aim:**

The objective of this study was to explore the genetic association of myeloperoxidase (*MPO*) gene polymorphisms with risk of Alzheimer's disease (AD).

**Methods:**

Blood samples were collected from 116 AD patients and 134 age and gender matched healthy individuals. Polymerase chain reaction-restriction fragment length polymorphism (PCR-RFLP) was utilized to confirm *MPO* polymorphisms in promoter region. Plasma concentration of MPO was detected by enzyme-linked immuno sorbent assay. Genotype distributions of *MPO* polymorphisms were compared by χ^2^ test between the two groups. The status of linkage disequilibrium between *MPO* two polymorphisms was detected using Haploview. MPO concentrations were analyzed by non-parametric test.

**Results:**

*MPO* rs2333227 polymorphism was positively associated with AD risk, especially under the AA+GA vs. GG and A vs. G genetic models (*P*=0.042, OR=1.719, 95%CI=1.017-2.906; *P*=0.041, OR=1.582, 95%CI=1.016-2.463). While, rs34097845 polymorphism significantly decreased the risk of AD, particularly GA and AA+GA genotypes (*P*=0.048, OR=0.555, 95%CI=0.308-0.998; *P*=0.042, OR=0.552, 95%CI=0.310-0.983). In addition, rs2333227 genotypes affected the plasma concentration of MPO. But for rs34097845 polymorphism, only GA genotype exhibited significant association with MPO concentration.

**Conclusion:**

Polymorphisms in the promoter region of *MPO* distinctly contribute to AD risk possibly through regulating MPO concentration. Present results should be confirmed by further studies.

## INTRODUCTION

Alzheimer's disease (AD) is one of the most common neurodegenerative diseases in old people. It is a long lasting disorder with irreversible process [1]. Its incidence is increasing with age, and the symptoms are becoming serious over the time [2]. The clinical symptoms of AD are characterized by short-term memory loss, language disorder, and behavioural issues [3]. The therapy and nursing of AD cause heavy economic burden and life stress to patients’ family and the society [4, 5]. However, the etiology of AD is not well understood. A lot of studies indicate that the major pathological characteristics of AD include abnormal deposition of amyloid beta (Aβ) and tau protein in brain, the loss of synapses and neurons, and the neurofibrillary tangles [6–9]. Besides, other researches find some defects may be also involved in the development of AD, such as vasculopathy, lipidosis, autophagy and lysosomal defects, etc [10–13]. AD is a complex disease which is regulated by the genetic and environmental factors [14]. It is generally considered that the genetic factors, especially the key genes associated with the pathological characteristics, play a crucial role in AD [15].

Myeloperoxidase (MPO), a heme protein, is abundant in neutrophil granulocytes and monocytes [16]. MPO has the activity of peroxidase and lysosomal enzyme [17]. MPO mediates the lipid metabolism [18], inflammatory responses [19], tissue damages [20], lysosomal pathway [21], and oxidative damage [22]. Abnormal expression of MPO may result in various disorders. It has been indicated that MPO was potentially associated with several neurodegenerative diseases [23]. For example, Tzikas et al. found that the plasma level of MPO was significantly higher in AD patients than that in healthy controls [24]. In human, MPO protein is encoded by *MPO* gene which is located in 17q23.1. Polymorphisms in *MPO* gene could alter the structure or expression level of the corresponding protein [25]. For instance, rs2333227 (-463A/G) polymorphism is located at the promoter region of *MPO* gene, which can bind with SP1 site. Alterations in the locus (463A) may influence the transcription activity of the gene, thus impacting the level of *MPO* [26, 27]. Rs34097845 (-129 G/A) is another SNP located at the promoter region of MPO gene, which has been proved to be associated with myocardial infarction risk [28]. However, the effects of these two polymorphisms on risk of AD remained unclear.

A case-control study was performed to investigate the association of *MPO* rs2333227 (-463A/G) and rs34097845 (-129 G/A) with AD risk in Chinese Han population.

## RESULTS

### The basic characteristics of subjects in this study

The demographic information of subjects in the case and control groups was showed in Table 1. The case group included 46 males and 70 females with the mean age of 67.39±9.18 years old, while there were 56 males and 78 females with the mean age of 68.17±8.97 in healthy control group. The case and control groups were matched in gender and age (*P*>0.05 for both). In this study, the age of onset in AD patients was 63.48±4.51 years old. Neither body mass index (BMI) nor tobacco consumption was the influence factor of AD risk in this population (*P*=0.163 and 0.169 respectively). However, the percentage of drinker in AD group was obviously higher than that in controls (37.07% vs. 24.63%, *P*=0.033), indicating drinking might be a risk factor of AD. In AD group, 8.62% cases were diabetes patients, and the ratio in control group was 4.48%, but there was no significant difference (*P*=0.182). The distribution of hypertension was significantly different between AD and healthy groups (*P*=0.037).

**Table 1:** The demographic characteristics of subjects in the case and control groups

Characteristics	Case, n=116	Control, n=134	*P*
Gender			
Male/female	46/70	56/78	0.732
Age (year)			
Mean age	67.39±9.18	68.17±8.97	0.654
Age of onset	63.48±4.51	-	
Body mass index (kg/m^2^)			
Mean value	25.32±6.35	24.57±5.51	0.163
Tobacco consumption (%)			
Yes	38 (32.76)	32 (23.88)	0.119
Drinking (%)			
Yes	43 (37.07)	33 (24.63)	0.033
Diabetes (%)			
Yes	10 (8.62)	6 (4.48)	0.182
Hypertension (%)			
Yes	23 (19.83)	14 (10.45)	0.037

### HWE test

Distributions of rs2333227 and rs34097845 SNPs genotypes and alleles were confirmed to HWE test both in cases and controls (Table 2, *P*>0.05 for all), indicating the good representativeness of our study population.

**Table 2:** Association between *MPO* polymorphisms and AD risk

Genotype/allele	Case n=116(%)	Control n=134(%)	Models	*P*	OR(95%CI)
rs2333227					
GG	68(58.62)	95(70.90)	AA vs. GG	0.262	1.956(0.596-6.423)
GA	41(35.34)	34(25.37)	GA vs. GG	0.063	1.685(0.971-2.923)
AA	7(6.03)	5(3.73)	AA+GA vs. GG	0.042	1.719(1.017-2.906)
G	177(76.29)	224(83.58)	AA vs. GA+GG	0.396	1.657(0.511-5.369)
A	55(23.71)	44(16.42)	A vs. G	0.041	1.582(1.016-2.463)
*P*_HWE_	0.805	0.382			
rs34097845					
GG	92(79.31)	91(67.91)	AA vs. GG	0.623	0.495(0.044-5.550)
GA	23(19.83)	41(30.60)	GA vs. GG	0.048	0.555(0.308-0.998)
AA	1(0.86)	2(1.49)	AA+GA vs. GG	0.042	0.552(0.310-0.983)
G	207(89.22)	223(83.21)	AA vs. GA+GG	1.0	0.574(0.051-6.412)
A	25(10.78)	45(16.79)	A vs. G	0.053	0.598(0.354-1.011)
*P*_HWE_	0.738	0.272			

Note: HWE: Hardy-Weinberg equilibrium.

### Association between *MPO* polymorphisms and AD risk

The sequenced results showed the data between PCR-RFLP and sequencing analyses were consistent. Thus, the genotyping results for *MPO* polymorphisms obtained via PCR-RFLP were reliable.

The frequencies of GA and AA genotypes in rs2333227 polymorphism were significantly higher in AD patients than that in healthy controls (*P*>0.05 for both). In comparison with GG genotype, AA and GA genotypes had no significant association with the risk of AD (Table 2, *P*>0.05 for both). Besides, AA+GA genotypes was associated with increased risk of AD (*P*=0.042, OR=1.719, 95%CI=1.017-2.906). Meanwhile, A allele was frequently observed in case group, suggesting that A allele might be significantly related to AD risk (*P*=0.041, OR=1.582, 95%CI=1.016-2.463). For the rs34097845, GA frequencies between AD patients and the controls were significantly different (*P*=0.048). GA genotype was correlated to decreased risk of AD (OR=0.555, 95%CI=0.308-0.998). Additionally, rs34097845 also showed obvious association with decreased risk of AD under AA+GA vs. GG model (*P*=0.042, OR=0.552, 95%CI=0.310-0.983). Lower frequency of A allele was discovered in AD patients, however, it had no significant association with AD risk (*P*>0.05).

### The analysis of haplotype consisted of *MPO* rs2333227, rs34097845 in AD risk

The strong linkage disequilibrium was found between rs2333227 and rs34097845 polymorphisms, and haplotypes G-G, A-G and G-A were detected in this study population, but not G-A haplotpye. The frequencies of haplotypes in AD patients and the controls were showed in Table 3 and the data were 65.52%, 23.71%, 10.77% in the case group and 66.79%, 16.42%, 16.79% in the control group respectively. But there was no significant difference between the two groups in haplotype frequency, so they did not show obvious association with AD risk.

**Table 3:** The haplotype analysis of *MPO* polymorphisms in AD risk

rs2333227-rs34097845	Haplotype		*P*	OR (95%CI)
	Case, 2n=232 (%)	Control, 2n=268 (%)		
G-G	152 (65.52)	179 (66.79)	-	Ref.
A-G	55 (23.71)	44 (16.42)	0.092	1.472 (0.937-2.312)
G-A	25 (10.77)	45 (16.79)	0.118	0.654 (0.383-1.117)

### *MPO* polymorphisms influenced the plasma MPO concentration

Plasma concentration of MPO were 326.50±52.28 and 276.01±24.87 μg/L respectively in AD patients and healthy controls. MPO concentration in different genotypes were listed in Table 4. In case group, AA genotype of rs2333227 had lower MPO concentration than GG and GA genotypes (*P*<0.01), GA genotype also exhibited low MPO concentration compared with the GG genotype (*P*<0.01). In the control group, GA and AA genotype carriers respectively had lower MPO concentration (*P*<0.01). Significant differences existed in the MPO concentration between the case and control groups respectively in GA and GG genotypes (*P*<0.01), but AA genotype carriers in case and control groups exhibited similar level of MPO. Rs34097845 GA genotype carriers both in AD and healthy control groups had higher MPO concentrations than those GG genotype carriers (*P*<0.01). Significant differences of MPO concentration were observed in GG and GA genotype carriers between the cases and controls. AA genotype of rs34097845 SNP had no distinct influence on plasma MPO concentration.

**Table 4:** Influences of *MPO* polymorphisms for plasma MPO concentration

Genotypes	Control (mean±SD, μg/L)	Case (mean±SD, μg/L)
Total	276.01±24.87	326.50±52.28^&^
rs2333227		
GG	283.03±17.36	350.88±42.71^&^
GA	262.71±27.62^*^	299.22±40.83^*&^
AA	233.2±47.19^*^	249.43±46.49^*#^
rs34097845		
GG	271.16±20.06	317.60±51.80^&^
GA	286.98±31.13^*^	361.65±39.56^* &^
AA	272±12.73	337

Notes: ^*^, compared with major allele homozygote *P*<0.01; ^#^, compared with heterozygote *P*<0.01; ^&^, compared with controls *P*<0.01. According to a Bonferroni correction, *P*<0.05/2=0.025 was the significant level.

## DISCUSSION

AD is a chronic neurodegenerative disease, without effective treatment. Thus, prevention is pivotal for the patients. Until now, the etiology of AD still can not be well understood. The initiation of AD is a consequence of synergistic effect of multiple factors, including genetic and environmental factors. Up to now, a number of risk factors are confirmed for AD, such as obesity, depression, tobacco consumption, drinking abuse, low degree of education, lack of exercise, hypertension, etc. However, in clinic, only a small part of people expose to these factors will finally develop AD, suggesting individual susceptibility to AD. Growing evidences have demonstrated that genetic alterations may be responsible for the individual susceptibility to AD [29]. In addition to β-amyloid (Aβ) and Tau protein, there are a large number of genetic molecules which may take part in progression of AD. To investigate the AD-related SNPs may provide a new insight into the etiology of the disease [30].

In present study, we investigated the associated of *MPO* polymorphisms with risk of AD in Chinese Han population. The results indicated that rs2333227 SNP was obviously associated with AD risk under five genetic models. In AA+GA vs. GG model, rs2333227 SNP obviously promoted 1.719 times risk of AD. Besides, 1.582 times risk of AD was presented by the carriage of rs2333227 A allele, compared with G allele. Under other genetic models, rs2333227 SNP slightly elevated the risk of AD. This result was accorded with previous studies [31–33]. But Combarros and Usui had reported that rs2333227 SNP was not related to AD risk [34, 35]. Negative association was discovered between rs34097845 SNP and AD risk. Compared with GG genotype, GA genotype and AA+GA genotypes carriers respectively were 0.555 and 0.552 times risk of AD. This was the first study to focus on the association between rs34097845 SNP and AD risk in Chinese Han population. But in the association study of coronary artery disease (CAD), rs34097845 exhibited no association with CAD severity and it did not affect serum MPO activity [36].

MPO concentration in plasma was significantly different between AD patients and healthy controls [24]. In current study, obviously high MPO concentration was observed in AD patients, this result was consistent with previous study [24]. It has been reported that rs2333227 A allele significantly reduced the transcriptional level of MPO [37], thus we speculated that this SNP might be related to the plasma concentration of MPO. Then we found that MPO concentration was lowest in rs2333227 AA genotype carriers, and GG genotype carriers had the highest concentration. Moreover, AD patients with GG and GA genotypes respectively had higher MPO concentration than that in controls, but not AA genotype. A previous study demonstrated that rs2333227 G allele enhanced MPO expression in astrocytes [38]. Rudolph and colleagues found that rs2333227 AA genotype was correlated to the decreased MPO expression in patients with impaired left ventricular function [37]. We also analyzed the correlation between rs34097845 genotypes and plasma MPO concentration. This results demonstrated that A allele of rs34097845 slightly decreased the MPO concentration in plasma. Based on the data, we hypothesized that rs2333227 and rs34097845 might be involved in etiology of AD through regulating the transcriptional activity of the gene. It has been reported that abnormal expression of MPO in astrocytes could result in a specific pattern of phospholipid peroxidation and neuronal dysfunction, thus contributing to AD [38]. However, the molecular mechanisms underlying the functional roles of MPO polymorphisms in AD still remained poorly known. Further investigations will be required.

In addition, the haplotype analysis was also performed in our study. Unfortunately, we did not observe obvious association between MPO haplotype and AD risk based on our study subjects. Several reasons might be responsible for the results. First, the relatively small sample size might be responsible for the abnormal results. Second, the inappropriate reference might be a key cause of the unusual results. G allele of rs2333227 SNP was a relatively protective factor for AD, while G allele of rs34097845 polymorphism could increase the risk of AD. Thus, G-G might be not an appropriate reference in haplotype analysis of *MPO* polymorphisms in AD. The analysis of haplotype consisted of MPO rs2333227, rs34097845 in AD risk needed further investigations.

*MPO* polymorphisms in the promoter region are significantly associated with AD risk via regulating MPO expression in Chinese Han population. Nevertheless, several limitations exists in current study. The sample size is not large enough and the study group only includes one Chinese Han population. Moreover, the interaction of *MPO* polymorphisms with other factors such as environmental factors was not considered. In view of these reasons, well designed studies with large sample size and multiple populations are necessary to certify the role of *MPO* promoter polymorphisms in AD in the future.

## MATERIALS AND METHODS

### Subjects

Between 2014 January and 2016 January, 116 AD patients and 134 healthy controls were recruited from Tianjin Huanhu Hospital as the case and control groups respectively. All subjects were Chinese Han population without any blood connection. AD patients were diagnosed by two pathologists according to the diagnosis criteria [39]. Healthy controls were matched with the cases in age and gender. Any person who had other neurodegenerative diseases, vascular diseases or systemic diseases would be omitted from this study.

This case-control study was approved by the Ethnic Committee of Tianjin Huanhu Hospital. All of the participants signed the written informed consents.

### Sample collection

A total of 2 ml fasting venous blood was collected from each subject in the morning using blood collection tube with EDTA. Blood samples were centrifuged into leukocytes and plasma, and then stored at -80°C.

### Genotyping for *MPO* polymorphisms

Genomic DNA was extracted from leukocytes using the traditional phenol chloroform isoamyl alcohol method. *MPO* rs2333227 and rs34097845 polymorphisms were genotyped using polymerase chain reaction-restriction fragment length polymorphism (PCR-RFLP) method following previous studies [40]. Each test was repeated three times. Both of these two polymorphisms were determined GG, AG or AA genotype in selected subjects. 20% samples were randomly selected to confirm the genotyping results via the ABI 3700 DNA analyzer (Applied BioSystem, USA).

### Detection of MPO concentration

Plasma MPO concentration was detected by enzyme-linked immuno sorbent assay (ELISA) method using a human myeloperoxidase, MPO ELISA Kit (Jianglai Biotechnology, Shanghai) according to the manufacturer's instruction. Each test was performed in triplicate, and the average value was used for the final analysis.

### Statistical analysis

Hardy-Weinberg equilibrium (HWE) test was used to detect the representativeness of the subjects. Genotype distributions between cases and controls were compared by Chi square test or Fisher's exact test. Odds ratios (ORs) with 95% confidence intervals (CIs) were utilized to estimate the relative risk of AD. The linkage disequilibrium of *MPO* two polymorphisms was analyzed by Haploview software. Plasma MPO concentration was assessed by non-parametric test (Mann-Whitney U test), meanwhile, the significant level was adjusted by Bonferroni method. All the calculations were performed by PASW 18.0. *P*<0.05 was considered as significant.
